# Service Providers’ Experiences Engaging Indigenous Fathers and Two-Spirit Parents With Young Children in Parenting Programs: An Interpretive Description

**DOI:** 10.1177/10497323251317426

**Published:** 2025-04-03

**Authors:** Amy L. Wright, Jessy Dame, Rachel VanEvery, Kate McGall, Ysabella Willett, Stephanie D. George, Bonnie Freeman, Jessica E. Pace, David Johnson, FOTNG Steering Committee

**Affiliations:** 1 Lawrence Bloomberg Faculty of Nursing, 7938 University of Toronto, Toronto, ON, Canada; 2 584047 Community Based Research Centre, Vancouver, BC, Canada; 3 3710 McMaster University, Hamilton, ON, Canada; 4 3688 Dalhousie University, Halifax, NS, Canada; 5 Indigenous Diabetes Health Circle, Thorold, ON, Canada; 6 7984 Toronto Metropolitan University, Toronto, ON, Canada; 7 Fathers of the Next Generation Steering Committee, ON, Canada

**Keywords:** Indigenous fathers, Indigenous parenting, programming, service providers, qualitative, Indigenous health research

## Abstract

Healthy parenting is critical to the health of children and is often supported by prenatal and parenting programs. While numerous parenting programs exist for Indigenous families with young children, most do not meet the needs of men or Two-Spirit people. Participation of Indigenous fathers is typically low, and little is known about how service providers can engage Two-Spirit parents. This study aims to understand the experiences of service providers who deliver programming for expectant and parenting Indigenous fathers and Two-Spirit people to better engage parents in supportive programming that more effectively meet their needs to be successful parents. A community-led approach, along with Thorne’s Interpretive Description methodology and the Two-Eyed Seeing framework, was used to understand the experiences of service providers delivering programs. Interviews and discussion groups were used to collect data concurrently with a collaborative approach to analysis. Findings are presented in a thematic summary comprised of four themes: (1) Relationality; (2) Role as Teacher; (3) Advocacy; and (4) Supporting Healing. The findings suggest that service providers should first build a trusting relationship with parents, provide activities, and use teaching strategies that more effectively engage men and Two-Spirit people in learning, while using a strengths-based and equitable approach. Findings demonstrate an urgent need to recruit men and Two-Spirit people as facilitators within organizations to deliver programming that is reflective of the clients they seek to serve.

## Introduction

Healthy parenting is pivotal during childhood, supporting a child’s healthy life trajectory (Moore et al., 2015; Priest et al., 2012). Parenting is often supported by early childhood development programs, including prenatal programs, which offer expectant parents education and support, enhancing parent–child relationships and promoting healthy pregnancies (Godin et al., 2015; Government of Canada, 2023a, 2023b, 2023c). In Canada, national funding policies support expectant and new parents in their parenting journey (Government of Canada, 2023a), while provincial programs typically offer prenatal programs through public health units by way of trained early childhood development specialists (Best Start Health Nexus, n.d.).

Indigenous-specific prenatal programming across Canada and within the province of Ontario, such as the Aboriginal Head Start and Indigenous Healthy Babies Healthy Children programs, is federally or provincially funded, and offers culturally safe parenting and early childhood development resources embedded with local culture and inclusive of local teachings and traditions (Government of Canada, 2024; Government of Ontario, 2024). Indigenous-specific parenting programs emphasize cultural practices as fundamental to healing, and culture is integrated throughout program content, inclusive of traditional Elder involvement, crafts, ceremonies, food, storytelling, and other cultural practices (Gerlach & Gignac, 2019; Macvean et al., 2017). Indigenous approaches to prenatal care are important as they diverge from settler practices, which typically prioritize women and infant health (Blanchet-Cohen et al., 2021). Indigenous child-rearing frameworks foster connections within the family and are inclusive of all parenting roles including those for Two-Spirit individuals (Blanchet-Cohen et al., 2021). Two-Spirit is a term used to refer to an Indigenous construct that encompasses sexuality, sex, and gender identity (Carrier et al., 2020; Dykhuizen et al., 2022; Pruden & Salway, 2020). Indigenous peoples may choose to identify as Two-Spirit or with other Western terms (e.g., lesbian, gay, transgender, queer, questioning, intersex, and asexual) (Dykhuizen et al., 2022). The historical and ongoing impacts of colonization continue to harm Indigenous peoples, including the loss of traditional parenting roles and beliefs associated with sexuality and gender (Hunt, 2015; Singer, 2015; Sylliboy, 2022; Thomas, 1997).

Despite having a more holistic approach, the literature describing Indigenous-specific parenting programs demonstrates a lack of engagement with Indigenous men and does not discuss the participation of Two-Spirit parents (Ball, 2010; Bottorff et al., 2019; Gerlach et al., 2017; Reilly & Rees, 2018). One explanation for the lack of engagement of men may stem from societal views that predominately position this type of care, such as labor, birth, and infant feeding, as the responsibility of mothers rather than fathers (Godin et al., 2015). Additionally, evidence suggests that these programs are not typically engaging for men, leading to low levels of participation and involvement (Bottorff et al., 2019; Wright et al., 2022). Little has been described about the needs of Two-Spirit parents and programming to support parenting; however, what is understood about the needs of Two-Spirit and other gender and sexually diverse parents suggests mainstream health promotional programming is often unsafe and not inclusive (Wright et al., 2024).

Literature suggests some ways that health promotional programs can be more enticing to men, which in turn may facilitate their engagement. In a study by Bottorff et al. (2019), Indigenous fathers suggested that male-led, father-friendly programming was a strategy that would promote their engagement in a smoking cessation program. Other studies have found individualized approaches, group-based activities, low-cost activities, prioritizing relationship building, and incorporating trained and talented facilitators who can quickly tailor content to the group’s needs are strategies to better engage men in health promotional parenting programs (Bottorff et al., 2019; Buston, 2018; Carlin et al., 2021; Jenkinson, 2016; Pfitzner et al., 2018). Programming may be more inclusive by using language and images depicting various family structures, through the consistent use of client and service provider pronouns, and by individualizing care by asking for preferences (Wright et al., 2024).

Service providers who function as facilitators of parenting programs play an integral role in designing, delivering, and encouraging the uptake of health promotional programming such as prenatal, newborn, nutritional, and parenting-focused programs, by Indigenous families. Not all service providers are licensed with a health professional or educational licensing body, though some are registered social workers, early childhood educators, traditional Indigenous midwives, or other professions. Some service providers providing programming at Indigenous-led organizations do not have formal education, but receive on-the-job training, and many have cultural lived experiences and knowledge relevant to their role and the clientele they serve. It is important to acknowledge that service providers function within a system deeply rooted in colonial legacies such as the Residential School Legacy and Sixties Scoop, resulting in a lasting distrust of social and child welfare services, and healthcare (Gerlach & Gignac, 2019). As such, service providers most effectively deliver programming when they have a thorough understanding of the historical and contemporary lived contexts impacting Indigenous peoples, and prioritize building trusting relationships with parents (Gerlach & Gignac, 2019). A culturally safe approach to service delivery starts with service providers recognizing their own positionality, acknowledging power relations and the socio-historical realities impacting Indigenous families (Churchill et al., 2017; Papps & Ramsden, 1996). Service providers who deliver culturally safe programming promote trust, empower families, and deliver holistic care, which at times means helping families to address compounding social concerns such as unemployment and housing insecurity (Gerlach & Gignac, 2019; Gerlach et al., 2017). This approach to service delivery helps foster engagement by facilitating a sense of parental control and safety.

Despite these important roles of service providers in Canada delivering parenting education and engaging parents in programming, how they effectively engage Indigenous men, fathers, and Two-Spirit parents in parenting programming is not well understood. This study intends to contribute to this knowledge gap, through understanding how service providers effectively engage Indigenous men and Two-Spirit parents in Indigenous-specific parenting programming for families with young children. This information is vital to supporting the effective delivery of existing parenting programs and to better meet the needs of Indigenous fathers and Two-Spirit parents.

## Methods

This study is part of the Fathers of the Next Generation project, a three-phased project originating from the experiences of numerous First Nation fathers and service providers who recognized the lack of father-focused parenting programming for new and expectant First Nations, Inuit, and Métis fathers and Two-Spirit parents. Phase 1 of the project consists of three qualitative studies which aim to understand the journey Indigenous men take to becoming fathers; the experiences and service needs of Two-Spirit parents as they transition to parenthood; and service providers’ perspectives of their role supporting fathers on their journey to parenthood. In phase 2, the understanding gained during phase 1 will inform the community-led design of parenting resources to support Indigenous men and Two-Spirit people in their transition to becoming parents. In phase 3, a pilot program of the resources will be implemented to evaluate their usefulness with Indigenous parents using community-identified indicators of success. This article describes the findings of a phase 1 study that focused on understanding the perspectives of service providers on their role supporting fathers and Two-Spirit parents on their journey to parenthood.

### Community-Led Design

The Fathers of the Next Generation project was designed using a community-led approach. An initial small group of community members from Six Nations of the Grant River spent a year speaking with fathers and service providers and co-developed a grant application for funding. Once funding was awarded, the group expanded to include representation of several Indigenous-led community organizations, including the Indigenous Diabetes Health Circle, Hamilton Regional Indian Centre, Six Nations Social Services, and the Six Nations Birthing Centre. The resulting Steering Committee consists of approximately 20 individuals (numbers fluctuate with staffing changes) including First Nations fathers and Two-Spirit parents, service providers, Indigenous scholars, and researchers of settler ancestry, and includes representation from numerous First Nations and Métis communities. The Steering Committee oversaw the design of all project components and participated in all project decisions. Ethics approval was granted by the University of Toronto Research Ethics Board and the Six Nations Research Ethics Committee. In keeping with OCAP® principles, data is owned by the Indigenous-led organizations and community partners and is stored locally by these organizations for access by community members following study completion.

### Situating the Authors

It is important we situate ourselves to the land on which we reside and our cultural identities to provide an understanding of our collective ways of knowing and perspectives. The lead author (ALW) is of European Irish and Scottish ancestry and resides on the traditional territories of the Mississauga and Haudenosaunee. She is a researcher who has worked with local Indigenous-led organizations and communities for more than 10 years, supporting the needs of the community through research. JD is Two-Spirit and Métis registered nurse, who is the Director of Two-Spirit Health at the Community-Based Research Centre in British Columbia. RV is Grand River Mohawk, currently a PhD candidate in the Department of Health, Aging, and Society at McMaster University, with degrees in nursing and public health. KM is of settler ancestry and contributed as a research assistant on the article. YW is a Mi’kmaw/settler woman from Wasoqopa’q (Acadia) First Nation located in Mi’kma’ki and currently an MD candidate at Dalhousie University. SDG is Oneida from Six Nations of the Grand River and French Canadian. She is an Ogwehoweh midwife and board-certified lactation consultant, and an instructor and MSc student at McMaster University. BF is Algonquin/Mohawk from Six Nations of the Grand River and is an Indigenous scholar in the School of Social Work and Indigenous Studies at McMaster University. Before her academic life, she worked with her community as a social service provider. JEP is Knowledge Program Manager at the Indigenous Diabetes Health Circle. She holds a PhD in the Anthropology of Health focused on community-based, participatory research with Indigenous communities. DJ is a First Nation father and nurse from Six Nations of the Grand River. Finally, the Fathers of the Next Generation Steering Committee contributed throughout this project, guiding its design and implementation.

### Theoretical Framework

The Two-Eyed Seeing framework is applied to our work, understanding how Indigenous ways of knowing and those of settler academics can work together, recognizing the utility of both Indigenous and Western ways of knowing (eyes) on viewing the world and solving problems (Bartlett et al., 2012). Our application of Two-Eyed Seeing is incorporated along with the 4Rs of research (Kirkness & Barnhardt, 1991); this project has been determined as *relevant* to parents from the Six Nations of the Grand River and the greater Hamilton area as well as by several local Indigenous-led community organizations. The Steering Committee prioritizes *respectful* collaboration and dialogue, recognizing our *responsibility* to ensure Indigenous Fathers and Two-Spirit parents’ ways of understanding the world are emphasized throughout our work in strengths-based ways that do not contribute to stereotypes or lead to harm. We thoughtfully consider how we are *reciprocal* in our work, by compensating participants and Steering Committee members for their time, building research skills and capacity among community partners through hiring and engaging community members to support the work (e.g., data analysis and manuscript writing), hiring local Indigenous caterers and hosting events at local venues, and making clear our commitment to the wellness of all those who participate in the study by explicitly outlining risks and benefits while also inviting their participation in the project.

### Methodology

Interpretive description methodology was used to support our understanding of the perspectives of service providers on their role in supporting the journey to fatherhood of Indigenous men and fathers (Thorne, 2016). Interpretive description is a pragmatic approach to qualitative research that recognizes and values the expertise of clinicians and service providers as essential to creating meaningful change at a service delivery level (Thorne, 2016). Interpretative description stems from constructivism, which recognizes the influence of context and intersecting factors on people’s perceptions and construction of reality (Thorne, 2016). The approach is flexible in nature, allowing the researcher to choose methods that align best with the research question to solve problems. In this way, we used interpretive description integrated with a Two-Eyed Seeing (Bartlett et al., 2012) approach to integrate Indigenous ways of knowing throughout the research process, while privileging the voices and expertise of Indigenous participants, research team, and Steering Committee members. This study explored the following research questions with service providers: (1) How do service providers support Indigenous men and Two-Spirit parents? (2) What needs do Indigenous men and Two-Spirit people have as they become parents?

### Setting and Sample

Inclusion criteria for this study included participants acting in a service provider role with at least 3 months of experience facilitating parenting-related programs for Indigenous fathers or Two-Spirit parents, expectant fathers or Two-Spirit people, and their families in programs delivered within the greater Hamilton, Niagara, Brantford, or Six Nations of the Grant River areas of Southern Ontario. Parenting-related programs could include any programs that involved parents and children, including prenatal programs, nutritional programs, programs focusing on the care of newborns or young children, and programs that focused on parent communication and interaction with their children. Purposeful sampling techniques based on inclusion criteria were used to recruit service providers with relevant experience using email, flyers, social media posts, and word of mouth.

### Data Collection

Eligible participants were enrolled in the study after providing informed verbal consent to either the research coordinator, interviewer, or group facilitator, prior to participating. Data were collected using discussion groups and interviews using semi-structured interview guides, facilitated by a member of the team without pre-existing relationships with the participants, and lasting 90–120 minutes. Discussion groups typically took place in person at one of our community partner organizations, while interviews took place virtually using Zoom.

The interview questions were designed with a subgroup of the Steering Committee using the *Indigenous Wellness Framework* (Health Canada, 2015). The framework takes a holistic approach to wellness, inclusive of spiritual, emotional, physical, and mental components, along with their associated outcomes: hope, belonging, purpose, and meaning (Health Canada, 2015). Interview questions were developed using these wellness components and their outcomes, to ensure a holistic definition of wellness was used and to elicit expertise that could be used to inform the design of a parenting program in phase 2 of the project. Some examples of interview questions include: *Can you share a bit about the types of programs or services you provide to Indigenous men/Two-Spirit people who are becoming parents or are already parents?; Can you share a positive or success story you’ve had in your role supporting fathers or Two-Spirit parents?; and Can you describe how you integrate Indigenous knowledge and culture into the supports you provide to fathers and Two-Spirit parents?*

Given that most discussion groups were attended by service providers who were also employees of the host site, only a few demographic details were collected from participants, including Indigenous identity and years of service. This was to promote the anonymity of the dataset, helping participants feel more comfortable being honest with their responses because their data would be less identifiable by their employers. The discussion groups and interviews were audio-recorded, transcribed to Microsoft Word, and de-identified by one member of the research team.

### Data Analysis

Data collection and analysis occurred concurrently and progressed through several iterations of coding and thematic analysis using structural and axial coding strategies as suggested by Saldana (2016). In keeping with our Two-Eyed Seeing approach, two First Nations research assistants, the lead author, and the research coordinator conducted analysis separately, each applying their own worldviews to their interpretations of the data. Then, they discussed together and came to consensus on a single set of themes which were shared with the Steering Committee for feedback. One of the research assistants and the lead author incorporated the feedback from the Steering Committee into a second iteration of analysis. Final feedback from the Steering Committee was sought and incorporated into the results presented here.

## Results

The findings of this paper are derived from the experiences of 23 service providers and reflect the merging of Western and Indigenous worldviews that occurred during data analysis while deferring to Indigenous perspectives and ways of knowing to identify cultural nuances and meanings in the results. Four participants completed a one-on-one interview, and 19 other participants engaged in one of three discussion groups. We returned to four participants to conduct second interviews, to fill gaps in knowledge recognized by the research team during data analysis related to strategies to effectively engage parents in programming. Nine participants provided parenting programming in organizations in a First Nation community, and the remaining provided services in Indigenous-led organizations in urban areas. One service provider was of settler ancestry and the others self-identified as First Nations. The number of years each service provider served in their current role ranged from 8 months to 25 years. Three service providers had experience caring for Two-Spirit clients in the programming they routinely offer for Indigenous families, and two of these individuals had delivered programming specifically for Two-Spirit people. As such, the results primarily describe service providers’ experiences engaging men and parents who identify as fathers, although all service providers spoke to providing programming that was welcoming and safe for Two-Spirit parents and their families. A thematic summary of the results from data collected with service providers is described in detail, consisting of four themes: (1) Relationality; (2) Role as Teacher; (3) Advocacy; and (4) Supporting Healing. Findings are presented using a strengths-based approach, as suggested by the Steering Committee.

### Relationality

Service providers’ commitment to a relational approach was evident by their need to establish a relationship with parents prior to initiating any program or service delivery and preceding any expectations that parents might be willing or ready to receive their support. Relationality consisted of two subthemes: (1) creating a safe space and (2) gaining trust.

#### Creating a Safe Space

Service providers created a safe space listening to their clients and building clients’ self-esteem and self-worth through their programming. One service provider shared how they create a safe space for men in their programs:

It’s making it safe enough for men. And it doesn’t take much for a man to feel unsafe in these services no matter how tough he looks. No matter how tough wording that he has, just the fact that they’re willing to come into a service usually that tells me that toughness is just the exterior. There’s something really vulnerable inside. That’s why that toughness is there. So being careful and gentle with these tough guys, which is a good portion of the people who are coming into service, that that’s a huge thing.

Another service provider shared that when they intentionally include fathers in prenatal visits, a space typically associated with mothers, men start to open up and recognize their new role as a parent.

I think that our men are very shy. They really are skeptical of who they trust to talk to. There’s a lot of gaps that are there. Because when I go and see them, if they’re there, I’ll include them in the conversation. Because usually they’ll sit back and they think, oh, this is just for the mom and baby. So, I’ll say, “So how, how’s that feel like to be a dad?” And so, I try to get them involved in the ... just little, small, like “Who me?” And it was like they’re amazed that they’re being asked a question and sometimes it, reality hits them right there and then that they are a dad.

#### Gaining Trust

Through establishing a safe space where men feel listened to and validated as equal parents alongside their partners, service providers can start to build trusting relationships with parents. This priority stems from a genuine love for the community they are working with, being persistent and reliable in their approach with men, and having authentic lived experiences.

A service provider shared their reasoning for the importance of first developing trust with their clients before their advice and health teaching can be heard:

… once you develop trust …they’re willing to give more space to hear that person out, and what they may say. So, it’s all a real development that relationship first has to develop before I can start saying this is what you need to do as a parent.

Service providers demonstrated genuine care for the community they were working with, which laid the foundation for gaining trust with clients. One member shared how a genuine love for the community is essential to being effective:

I tell my students; you have to love these people. You have to love them. Not just, oh I enjoy this job. No, you have to love them because this is their life that you’re working with. And so if you don’t like them, you’re not going to help them.

Another key element of gaining trust with their clients was being reliable. Service providers shared how important it is to follow through. One participant shared how being available for clients when they said they would be is the same as being honest or truthful with them:

But forever be honest, forever tell the truth … if you’re going to let them know, “Hey, I’m available for you here or there and the other,” then also too, make sure that you’re available and make sure that you can always provide that availability.

Service providers having authentic lived experiences as a parent was another important element to gaining trust with clients. Service providers with lived experiences felt they more closely related to their clients and were better able to respond and meet their needs:

From my experiences having someone who’s gone through it or is going through it, helping me through that makes it that much more relatable. I can actually feel as though there is that connection, and this person is speaking from experience.

Having men speak at or facilitate programming was also viewed as important to being able to engage fathers in programming: “Male speakers are really important. They’re [fathers] more likely to come if there’s a respected traditional person that’s going to speak.” Yet, finding men to participate in these roles was very challenging and most service providers were women.

### Role as Teacher

Service provider participants connected intimately with their role as teacher. This theme consisted of three subthemes: *client-oriented programming, facilitating service connections*, and *teaching life skills along the journey to parenthood*.

#### Client-Oriented Programming

Most service providers shared the challenge of engaging men and Two-Spirit parents in programming. Those who had success, did so by providing client-oriented activity-based programming such as drumming, offering food, holding activities outdoors on the land, incorporating fire, building cradle-boards or medicine bags, incorporating ceremony, and at times facilitating opportunities for men to interact with their children. One service provider shared how activities that encourage men to be active foster their engagement in programming:

… the hands-on stuff really seems to work with our guys. So, if they had, say for example, something that they were doing with their hands and then they just start talking and they kind of have your things that you want to talk about. You can bring them out during that workshop, whatever it is. And then sometimes they’ll talk a little bit more. And food. You got to feed them.

Another service provider offered their experience with a drumming group:

And then the other thing is the men need to do something. They’re not ones to like, “Hey, let me sit and listen to you” … And then the drumming was … that they would learn … it was the teachings, but they were doing something while it was happening. And I think [they] like that. And the sweat too. It’s, they’re doing something as opposed to, what kind of traditionally they kind of sit and we talk to them …

By orienting programs and services to the interests of Indigenous fathers specifically, service providers improved their engagement with programming, enabling providers to better meet men’s needs. One service provider noted that land-based activities helped them to connect with a Two-Spirit parent and their children. However, there were no other examples offered by participants of how to better engage Two-Spirit parents. Instead, service providers strove to make all their programming welcoming to Two-Spirit parents by creating safe spaces.

#### Facilitating Service Connections

Service providers recognize they have limitations in their ability to meet all the needs that men and Two-Spirit parents have, but view connecting clients with other supports within their own organization, or with outside services, as an important facet of their role. Helping parents make connections is one way service providers deliver holistic care, recognizing parents have other needs such as housing, food, and employment support, and that a single service provider is unlikely to meet every need a client has.

… connecting them to, if need be, like justice, support advocacy. Connecting them with supports for food security. Connecting them to um prenatal and children’s programs that we have here, youth programs that we have here. Um connecting them to like community partners for assistance either be it mental, physical health needs for those individuals, and in any of those scenarios, like doing my best to make sure that those are safe places for them to connect with.

Service providers commonly spoke to the limitations facing their organizations in terms of the scope of support they can provide and the availability of funding to meet clients’ needs. Despite these limitations, service providers often go the extra mile to link clients with other providers and organizations that can meet the needs that they are unable to.

#### Teaching Life Skills on the Journey to Parenthood

Having built a solid foundation of trusting relationships with their clients through a relational approach to care, service providers teach valuable life skills and lessons to fathers and Two-Spirit parents throughout their journey to parenthood. First, many service providers recognized that parents were coming from different stages and experiences in life, so they would strive to meet them where they are and help them become independent. For example, one service provider described their compassionate approach to helping parents become independent of their services:

So, helping them to do those things on their own and again, reestablishing those abilities that they do have. But just step by step, again, meeting someone where they are, as opposed to where we want them to be. You want them to be able to empower themselves and to live that good life. But we’re all different stages along that, right?

Next, service providers shared important information and life skills they taught during pregnancy, about and during birth, and in the postpartum period. Most service providers spoke to their experiences related to men and fathers, but all strove to provide inclusive and individualized care to all parents, regardless of their gender identity. One service provider shared their thoughts about how they prepare expectant fathers to support their partner and build a strong relationship during pregnancy.

… To help them with their roles and responsibilities, understand their roles and responsibilities, understand what it is they need to bring in their bundles. And then also having those conversations with them that it’s not just your partner who’s pregnant, but you’re pregnant too, right? …

Participants noted the positive impact of father involvement early in the prenatal period and throughout the pregnancy. Particularly related to how men can impact the mother’s health and behaviors, one service provider shared her multi-pronged approach using midwifery knowledge and connection to cultural teachings.

I know we’ve all talked to dads and just tried to explain how maybe a pregnant person’s emotions are. Right? … This is how you could help. If they’re feeling really stressed and irritable, maybe to explain why that is. And how … even traditionally some of the teachings around that, that the men are supposed to be a pillow and just not, don’t fight back. This is her hormones … she needs to keep a good mind. That’s really important. And you don’t want to argue with her … Just know that that’s just something she’s going through right now. So just even how to support her, I guess, being pregnant.

Another midwife shared how teaching men to have patience is more important than learning skills such as swaddling a baby, which can still be taught, but is not as vital as having patience and love for their partner and newborn. Midwives shared that many of the fathers they work with want to learn how to deliver the welcome speech to the baby. The welcome speech for a newborn is often recited in the family’s Indigenous language and are the first words the newborn hears as they enter the world (Oneida Digital Media, 2009).

We talked about support is really a big, even there have been a lot of requests over the years for Dads wanting to know how to say the welcome speech in the language. Really wanting that information because that’s important to us is that that’s said right after the baby’s born. And I know that there’s been some Dads that have really worked hard and done a really good job. And it was really nice. So that, there’s a big demand for that.

This midwife shared how she is intentional about involving fathers in their newborn’s first bath, as it helps them bond with the baby:

… including them [father], and that I find is one of the most bonding moments ever is when they do that first baby bath together. It’s very bonding. I mean, by the end, they’re both crying because the baby’s crying. But that’s bonding in itself. Right?

In the early postpartum period, service providers spoke to how they encourage fathers to engage in skin to skin with their babies to promote bonding and building a trusting relationship with their newborn:

So it makes them a much more patient parent, which is great in the middle of the night when babies might be going through a growth spurt or whatever, and they’re being extra, what's the word? I don’t like the word needy, because in Western society, that makes it look really bad. But babies always have a need. And for infants that skin to skin contact fulfills one of those needs automatically because babies can’t talk. So just holding the baby skin to skin is a lot of good. And then I tell the dads that if mom’s away doing shopping or just out with friends, that baby already trusts you. So, if baby’s going to get upset, the baby already trusts that you know how to calm him down, and you trust yourself that you have a beautiful, intimate relationship with your baby so that the baby and you will be fine.

Another midwife shared how she reinforces with fathers how they can interact with their babies to help them grow and develop and continue to foster their bond with the baby.

… And then, I mean, I work with dads too a lot, getting them to read books when the babies on their lap. And even though some of the people, some families in our community, they, they’re because of residential schools are so afraid of any type of education and they, they’ve become emotionally distant. Just having a baby on the lap can seem foreign and nerve-wracking. So, teaching that intimacy and learning on the lap, why that’s so important … And if they want to be the language teachers, if you’re pushing a baby in a stroller, just teach them the colors, teach them the trees, go for hikes and teach them the medicines that they’ll find. Dads are absolutely needed by the infant in healthy ways.

As infants grow into toddlers, service providers continue to support fathers in learning new ways to encourage the healthy development of their children.

And it’s a difficult task for sure, how to raise their babies. Some of them is like, “How do you raise your babies? How do you communicate with your babies? You can’t just not stimulate them. They need that stimulation. They need that affection from you. You need to provide that stimulation for them.” So again, setting up just that time, spending quality time with your kids, how are you going to do that? Even if it’s sitting there coloring and having a conversation, not playing a game or such like that, just having that interaction, going to the park and playing with them.

In their role as teachers, service providers take a client-oriented approach to meet fathers and Two-Spirit parents where they are, rather than imposing programming on them that does not meet their needs. To better engage fathers, they prioritize activities that interest men, often including food and hands-on activities. Recognizing the limitations of their role, they persist in linking fathers and Two-Spirit parents and their families with other services that can meet the needs their organizations cannot. In doing so, they promote fostering the parent’s eventual independence, helping them learn how to navigate community services so they can address their needs for the long-term regardless of the availability of the service provider. Throughout the journey to parenthood, service providers impart relevant information and life skills that support them as they anticipate and become parents. Many service providers care for parents and their families for many years, and as such, can continue to support their growth as parents, teaching new parenting concepts and skills as their children age.

### Advocacy

Having built a foundation based on trust with their clients, service providers felt a strong need to advocate for their clients, to support them to be treated fairly, and to promote healthy parenting. Service providers advocated for their client’s access to legal representation, for attending culturally safe programming alternatives to meet requirements set by child welfare services, for tangible resources, and to overcome stereotypes. One service provider whose role was to work with families involved with the courts shared their frustration with the lack of access to legal support:

There’s barriers every step of the way when it comes to the family court process … it’s really, really difficult oftentimes depending what income bracket you’re in, but to even get a legal aid certificate as a father who’s trying to get access, really difficult. And then if you’re not approved for a legal aid certificate, it’s like you have to do the whole court process on your own, which is so hard … And so while you’re trying to navigate this really difficult process that takes forever and you’re getting, you said getting beaten down along the way, in all that time, you’re not having access to your child … I’m building relationships with lawyers in the community who are like, “I want to help. I can’t take on legal aid clients right now, but if you have a question, call me” …

Another service provider shared how they prioritize their care for fathers involved with child welfare services by ensuring they have access to the supports they need to meet the requirements to regain access to their children.

… if the person is involved say with [child welfare services], then working with them to help them to address the issues that brought them in … and then advocate for them to get the access they need to the supports. And then also eventually getting their files closed …

This provider’s passion for social justice was palpable, and their frustration clearly fuels their desire to advocate for their clients. Recognizing how some of their clients struggle to access the necessities of life, service providers commonly spoke to their efforts to help address these needs by providing hot meals, transportation, formula, diapers, and support to find affordable housing, or apply for child benefits or a status card. While this participant thought providing a meal at programming was a simple act, this service is likely essential for many clients who face food insecurity.

… I think something really simple that we do when we run programming, is having meals provided. Meals provided, bus tickets. And that’s part of the building community and that’s part of building rapport. So, you’re coming and you’re knowing that you’re going to have a nice hot meal. So, I think that’s a really simple thing that we do within all of our programs at the centre. And sometimes the men that came in the early days just came to eat because that was their only home cooked meal.

The genuine love service providers have for their clients facilitates their drive to advocate for better access to services, fair and compassionate treatment, and access to necessities. While their role is supporting families and providing programming at their home organizations, they are all champions for social justice in their communities.

### Supporting Healing

The final theme describes the role of service providers in supporting fathers and Two-Spirit parents in their healing. For some, becoming a parent brings up past experiences of being parented, particularly memories of how they were parented by their own fathers. Some parents recognize the trauma they have experienced while others do not. Several service providers spoke about their ability to recognize trauma in their clients and how they help them move through past experiences to heal and be the best parent they can be. This service provider shared his approach to helping someone move past negative energy when he recognizes their unresolved trauma.

What is scary, though, is … just the unresolved trauma, and a person may not even be aware … I just really try to tell them in a, in a really loving, supportive way, that you know, we’re going to probably have to put a little bit more work in the average um person, in, into this to get us to a good starting point, because right now we’re, we’re, we’re not off to a good start. Um. But again, you can change that, and I’m here to help you. Um, and I’ll clean them off, I’ll uh, like with the medicines, um—Let them know there’s other medicines that they can, they can use to help on their journey to um, uh basically purge a lot of that negativity as well, if they choose to, if they choose not to, you know that’s, that’s their decision.

An Indigenous midwife shared how she helps fathers realize the importance of healthy relationships and connects them with supports to help them begin to heal so they can be a healthy parent for their newborn.

But Dads need to know that they have to have a happy and healthy relationship. If they don’t know, I help them to get resources. I can get them in touch with faith keepers or knowledge holders or get them into more western healing modalities that are run through the birth, not the birthing center, but through [band] council. Hopefully get them involved in some ceremonies because sometimes you need to release your trauma before you can be present for your own baby.

Learning how to communicate with one’s partner is also necessary to parent together in a healthy way, particularly as both parents are likely to bring past traumas into their relationship together and into their new role as parents.

… it’s like a two-way street too. You need to communicate … Because you are a couple and you’re in this together, and you have to figure out how you’re going to make ends meet by meeting each other’s needs and your parenting styles.

Supporting Indigenous men and Two-Spirit parents in their journey to parenthood meant helping them to unpack past experiences with their own parents, which was often focused on their relationships with their fathers, so that they could begin to heal from trauma. In doing so, they might be better prepared to enter their new role as a parent in a healthy way, able to communicate and work together with their partner, and bond and be present with their newborn.

## Discussion

Results reveal the experiences of service providers engaging and supporting Indigenous men and Two-Spirit people along their journey to parenthood. The findings fill an important knowledge gap concerning how to engage these parents in programming and meet their needs to promote healthy parenting and optimize their children’s development. Few studies describe how service providers engage fathers in parenting-related programming, and even fewer provide insights on how best to meet the needs of Indigenous fathers in these programs. Furthermore, we were unable to locate any studies that speak to how best to engage Two-Spirit parents in parenting programs focused on newborns and infants.

In keeping with our Two-Eyed Seeing approach, results reflect the strengths of service providers and Indigenous-led organizations, which highlight a relational approach to programming. Service providers spoke to the fundamental need to build trusting relationships with parents prior to engaging in program delivery. Using a relational approach to building trust, they created safe spaces where men and Two-Spirit people feel welcome to gather and interact with peers and other service providers. While participants spoke about their intention to create safe spaces throughout their programming, they did not share specific examples of how they create safe spaces for Two-Spirit people in programming offered to all parents and families. In a review of Two-Spirit and other gender and sexually diverse parents’ experiences using parenting supports for their children under the age of 5 years, many parents reflected on the lack of inclusive spaces, describing that typically parental programming spaces are dominated by heteronormative messaging, which made parents feel excluded (Wright et al., 2024). Additionally, parents shared they often act as educators for service providers, teaching them how to provide inclusive and welcoming spaces and how to best meet their needs (Wright et al., 2024). While the participants in the current study were aware of the need to offer inclusive and welcoming spaces and programming, it will be essential to consider the perspectives of their Two-Spirit clients to determine ways service providers can improve their care of Two-Spirit families.

Next, through intentional listening, participants learned about their client’s values and priorities, enabling them to use a client-oriented approach to best meet their needs. The literature demonstrates that using a client-oriented approach can effectively engage older men and adolescent fathers in health promotional programming (Anderson et al., 2016; McGirr et al., 2020), although neither of these studies included Indigenous participants. Other studies with Indigenous fathers have demonstrated that trained facilitators, particularly men, are essential to delivering client-oriented programming as they can adapt programs to local cultures to meet the unique needs of those participating (Bottorff et al., 2019; Carlin et al., 2021). While recognizing the importance of having men and Indigenous fathers participate as both facilitators and speakers in parenting programming directed at fathers, the service providers in our study spoke to struggling to hire men into these roles. Similarly, none of the participants identified as Two-Spirit or spoke of Two-Spirit staff who provide parenting programming at their organizations, suggesting there is also a need to recruit Two-Spirit service providers to better meet the needs of Two-Spirit parents who may wish to learn from others with similar perspectives.

Our collaborative approach to analysis, informed by the Two-Eyed Seeing framework, leveraged the experience and knowledge of our Steering Committee members, which allowed us to identify the meaning of culture and its impact on the programming preferences of men and Two-Spirit people. In their role as teachers, service providers recognized that men tend to prefer activity-based programming and enjoy programming in which they can interact with their children. Similarly, a service provider spoke about how providing a land-based activity at a local park helped them connect with a Two-Spirit parent and their children. Realizing the importance of being on the land, service providers endeavor to offer activities and events outdoors in spaces where clients can interact with nature and benefit from its healing effects. In a study aimed at promoting smoking cessation among Indigenous fathers in Western Canada, Bottroff et al. (2019) found similar findings, that men preferred programming integrated with outdoor activities such as sports and games in addition to traditional activities such as drumming and lacrosse.

Service providers facilitate parents becoming independent of their services, recognizing the importance of parents learning to make connections in the community on their own so they can meet their own needs and those of their family members without the assistance of a service provider. Yet despite this priority, service providers felt strongly about advocating for parents’ and families’ equitable access to services, fair treatment, and the necessities of life such as food, housing, and transportation while in their care. These priorities demonstrate how service providers take a strengths-based approach to the care of their clients, recognizing their clients’ capabilities when given the tools and resources to effectively develop their parenting roles and identities. In a study of racialized low-income fathers interacting with parenting programs, researchers recognized that low-income and racialized fathers tend to be stereotyped as “dead beat Dads,” and programming takes a deficit-based approach, underpinned by the belief that service providers must motivate fathers to be involved with their children (Randles, 2020). Instead, Randles (2020) calls for programming to take a strengths-based approach by providing the necessary resources and material goods to enable fathers to engage with their children. Jenkinson (2016) took a similar strengths-based approach to a parenting programming for fathers in Ireland, and found this emphasis enabled fathers’ resilience and self-esteem, as fathers who wish to create positive change in their lives do this by building on their strengths, not their limitations. The service providers in our study took a thoughtful approach to programming to build up parents’ self-esteem and enable them to develop positive parenting identities.

Finally, service providers recognized how becoming a parent made past unresolved trauma evident in their clients and aimed to help them address this using numerous avenues such as counselling, access to traditional healers, Elders, traditional medicines, and Western supports as required. Service providers believed that beginning to heal was important to a parent’s ability to parent their newborn in a healthy way, through developing communication skills, releasing negativity, and having a strong sense of their role in life. The literature echoes these findings, though studies focus on fathers and not Two-Spirit parents. Studies have found that for some Indigenous fathers, having children brings up trauma from their childhood and past relationships with their parents. In such cases, there is often a need to heal from intergenerational trauma resulting from colonization, the Residential School Legacy, and the Sixties Scoop, which have led to examples of unhealthy parenting due to a loss of traditional life, roles, and culture (Ball, 2009, 2013; Canuto et al., 2019; Dad Central Ontario, n.d.; Oster et al., 2018; Plunket, 2021; Stayin’ on Track, n.d.; Waddell et al., 2021; Women’s Health Clinical Support Programs Women and Newborn Health Service & Department of Health Government of Western Australia, 2015). Despite their invisibility in the literature, it is likely that Two-Spirit parents have similar needs for healing from trauma, which may be further compounded by additional negative experiences such as homophobia and transphobia (Dykhuizen et al., 2022). In our study, service providers were painfully aware of the challenges many of their clients experience, and how essential it is to begin to unpack and heal from trauma to be a healthy parent. In the future, researchers should ensure their work accounts for the needs of Indigenous men and fathers in addition to those who identify as Two-Spirit parents.

### Implications

Building trusting relationships is essential to being able to engage parents in future programming. However, this takes time, so organizations and funding bodies should enable service providers to provide events and activities that are inviting to Indigenous men and Two-Spirit parents, so that relationships can be established. Simultaneously, it is important that the numbers of Indigenous men and Two-Spirit people participating in programming are not made a priority until after sufficient time is allowed for relationship building, as they may be less likely to attend programs before these relationships are established. Second, service providers appear well-positioned to meet the needs of Indigenous men and Two-Spirit parents when they have similar life experiences, including identifying as men or Two-Spirit parents, respectively. Many of the participants’ places of work struggled to hire men and Two-Spirit service providers; thus, more emphasis should be placed on recruiting these important staff members, and further research can support how to best recruit people into these roles. Third, organizations are likely to have more success engaging men in their programming if they provide activity-based events and enable fathers with tangible resources such as food, and access to services. Finally, service providers must be equipped to identify clients with unresolved trauma and to support them in their healing.

These findings will be used to support phase 2 of the three-phased project, which includes co-designing parenting resources for Indigenous men and Two-Spirit people as they transition to parenthood. The insights shared by service providers have direct implications for designing an activity-oriented and relational approach to programming that is led by men and Two-Spirit facilitators, to facilitate building relationships with their clientele.

### Strengths and Limitations

This study is one of few that investigates the unique role of service providers in engaging Indigenous fathers and Two-Spirit parents to participate in parenting programs. The themes are informed by a large group of service providers who provide care in a variety of settings, including urban Indigenous Friendship Centres and in First Nations communities, and deliver programs that focus on families, healthcare, and social services. Despite the large sample size, most participants identify as women, yet the men who participated shared similar insights. The findings are also contextually bound to Southern Ontario, and, as such, the findings may not reflect the experiences of other Nations, or Métis and Inuit men and fathers, or those with other gender and sexual identities such as Two-Spirit parents. Like other studies, service providers in this study struggled to engage Indigenous fathers in services and programs. As such, strategies to engage men in programming may still be realized by research with individuals and organizations who report outstanding success at recruiting men to participate in services.

## Conclusions

This study provides insight into the role of service providers delivering programming and services to Indigenous men, fathers, and Two-Spirit parents in Ontario, Canada. Their unique perspectives shed light on how men and Two-Spirit parents might better be engaged in health promotional parent-focused programming, beginning with the need for a foundational relationship built on trust. Recognizing the unique ways men learn, service providers should incorporate hands-on activities into their programming to improve participation, inclusive of outdoor activities on the land, and cultural events. Service providers in this study demonstrated the importance of using a strengths-based and equitable approach, ensuring clients had access to the necessities and services required to implement and develop their roles as parents. Finally, further work to determine how to recruit and retain men and Two-Spirit identifying service providers is necessary to better engage men and Two-Spirit people in parenting programs.
